# Case Report: Detection and quantification of tumor cells in peripheral blood and ascitic fluid from a metastatic esophageal cancer patient using the CellSearch ^® ^technology

**DOI:** 10.12688/f1000research.3-12.v1

**Published:** 2014-01-15

**Authors:** Qian Tu, Marcelo De Carvalho Bittencourt, Huili Cai, Claire Bastien, Camille Lemarie-Delaunay, Marie C Bene, Gilbert C Faure

**Affiliations:** 1 CHU Nancy, Nancytomique, Laboratoire d’Immunologie, Pôle Laboratoires, Vandoeuvre lès Nancy, France and Université Henri Poincaré, Faculté de Médecine, EA4369 RHEM and UMR7039 CRAN-CNRS, Vandœuvre-lès-Nancy, France; 2 Service D’Anatomie et Cytologie Pathologiques, CHU Nancy-Brabois, Vandœuvre-lès-Nancy, France

## Abstract

Analysis of ascitic fluid should help to identify and characterize malignant cells in gastrointestinal cancer. However, despite a high specificity, the sensitivity of traditional ascitic fluid cytology remains insufficient, at around 60%. Since 2004 the CellSearch ^®^ technology has shown its advantages in the detection of circulating tumor cells (CTCs) in peripheral blood, which can perform an accurate diagnosis and molecular analysis at the same time. To our knowledge, no previous study has explored the potential utility of this technology for the detection and quantification of tumor cells in ascitic fluid samples. Herein we report a case of metastatic esophageal adenocarcinoma in a 70-year-old man presenting with dysphagia and a large amount of fluid in the peritoneal cavity. Analysis of a peripheral blood sample and ascites sample with the CellSearch ^® ^technology both revealed the presence of putative tumor cells that were positive for epithelial cell adhesion molecule (EpCAM) and cytokeratin (CK) expression. This study confirmed the hematogenous dissemination of esophageal cancer by the detection of circulating tumor cells in the peripheral blood, and is the first to demonstrate that tumor cells can be identified in ascitic fluid by using CellSearch ^®^ technology.

## Introduction

Esophageal cancer is the eighth most common carcinoma and the sixth leading cause of cancer-related death worldwide, with approximately 482,000 new cases and 400,000 associated deaths per year ^1^. There are two main histological types of esophageal cancer, namely esophageal squamous cell carcinoma (ESCC) and esophageal adenocarcinoma (EAC). Although more than 90% of malignant esophageal tumours are squamous cell carcinomas, the incidence of esophageal adenocarcinoma in Western countries has increased sharply in recent decades ^2^. Disease prognosis is strongly related to the stage at diagnosis ^3^, but EAC is commonly diagnosed at an advanced stage and palliative therapies are often the only treatment option, and therefore EAC has an extremely poor prognosis.

In particular, peritoneal dissemination is frequent in gastrointestinal cancer and analysis of the ascitic fluid should help to establish the etiology of ascites, and identify and characterize malignant cells. However, despite a high specificity, the sensitivity of ascitic fluid cytology remains insufficient, at around 60% ^4, 5^.

Recently, the detection of circulating tumor cells (CTCs) had an increasingly important role in the domain of oncological research. These cells have long been considered as a reflection of tumor aggressiveness, while hematogenous spreading of CTCs from a primary tumor could be an important step in the metastasis cascade, leading ultimately to the formation of overt metastases ^6^.

We herein report a case study of a 70-year-old man affected by esophageal adenocarcinoma with multiple metastases who was referred for dysphagia and abdominal effusion. At diagnosis, the patient underwent a peripheral blood and ascitic examination to detect and enumerate CTCs and determine their clinical significance.

## Case presentation

At the beginning of June 2012, a 70-year-old white male was referred to the emergency unit because of a progressive deterioration of general condition for 4 weeks, which included dysphagia, low-grade fever, anorexia, and a weight loss of 8 kg within 4 months. The patient had been suffering from chronic lymphocyte leukemia stage A for 4 years, with indolent evolution according to regular yearly follow-ups.

Initial physical examination was unremarkable except for a left infraclavicular adenopathy of 2 cm. A computed tomography scan identified a thickening of the lower esophagus, a hypodense lesion of the liver and a lymphatic mass in the mesenteric region.

**Figure 1.**
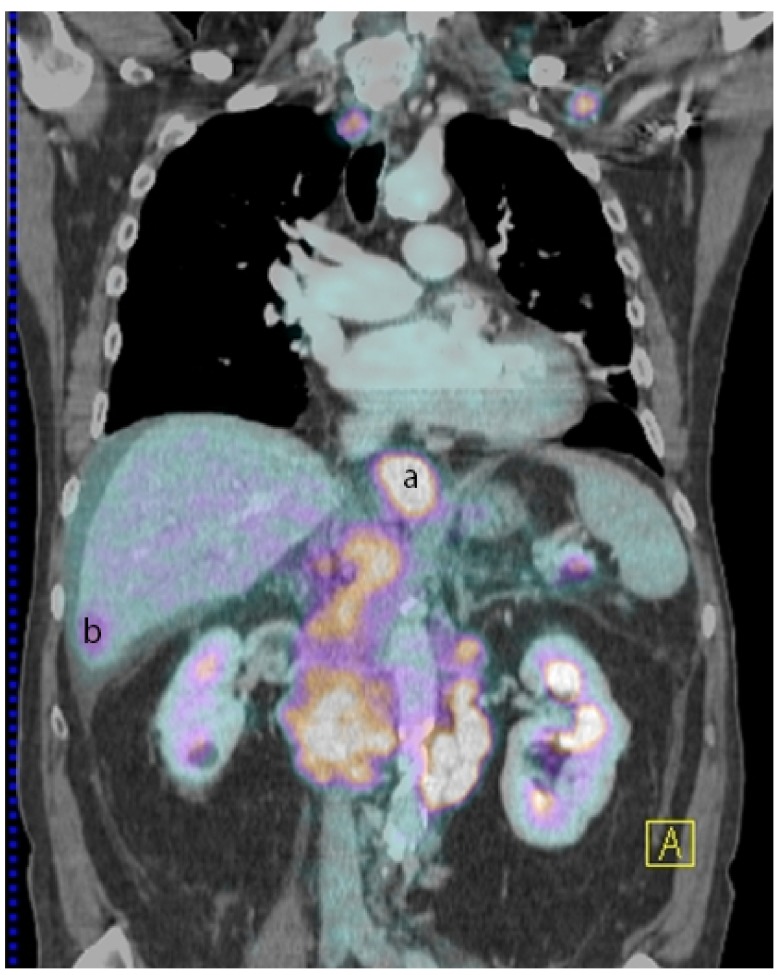
PET/CT, performed after administration of 311 MBq of Fludoxyglucose, showed increased activity in the lower oesophagus ( **a**), liver metastasis ( **b**) and pancreatic metastasis (not shown in the picture).

**Figure 2.**
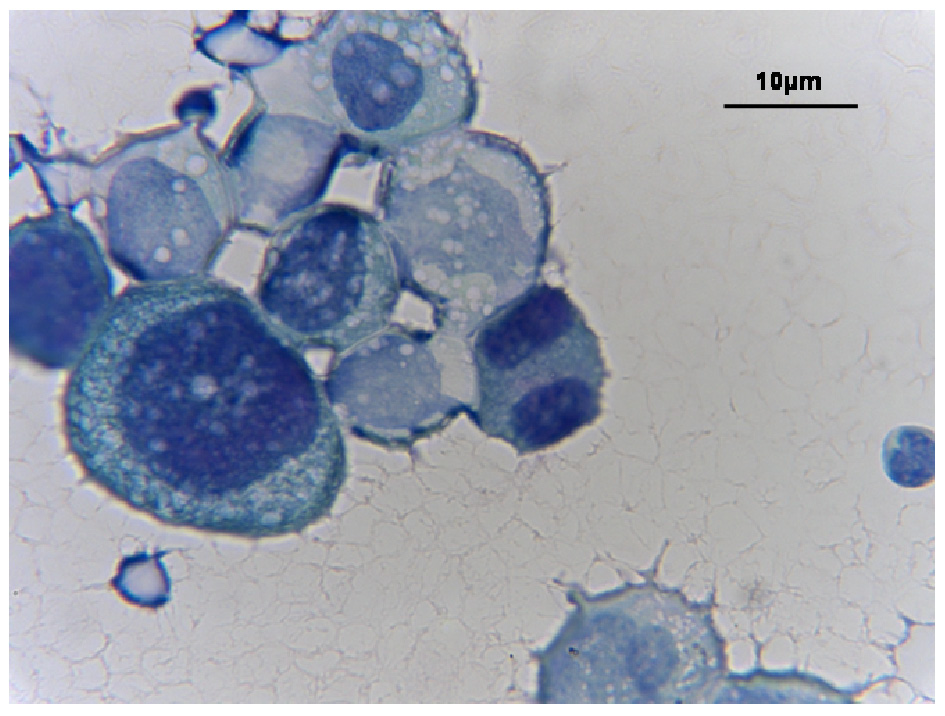
Tumor cells detected in the ascitic fluid of the patient with the ultimate diagnosis of esophageal cancer (Papanicolaou stain, ×1000).

The patient was then admitted to hospital for further investigations. During the period of hospitalization, the patient’s disease progressed quickly, with the appearance of massive ascites, jaundice, and respiratory difficulties. A PET/CT scan showed a thickening of the lower esophagus, supra- and sub-diaphragmatic adenomegalies and lesions in the liver, pancreas and lesser curvature of the stomach, all with hypermetabolic activities ( Figure 1). A gastroscopy showed a circumferential thickening, without stenosis in the inferior segment of the esophagus, bleeding easily on contact. A biopsy performed at this level demonstrated infiltration by a poorly differentiated adenocarcinoma. Fine needle aspiration biopsy of the liver lesion demonstrated the same tumor type with large cells of polygonal or cubic shape, and irregular hyperchromatic nuclei. These cells were also positive for cytokeratins CK7, 19, and 20 by a standard immunochemistry method. Both the first and second paracentesis revealed rare potential adenocarcinoma cells in clusters ( Figure 2), which were cytokeratins AE1/AE3, 7 and 19 positive.

**Figure 3.**
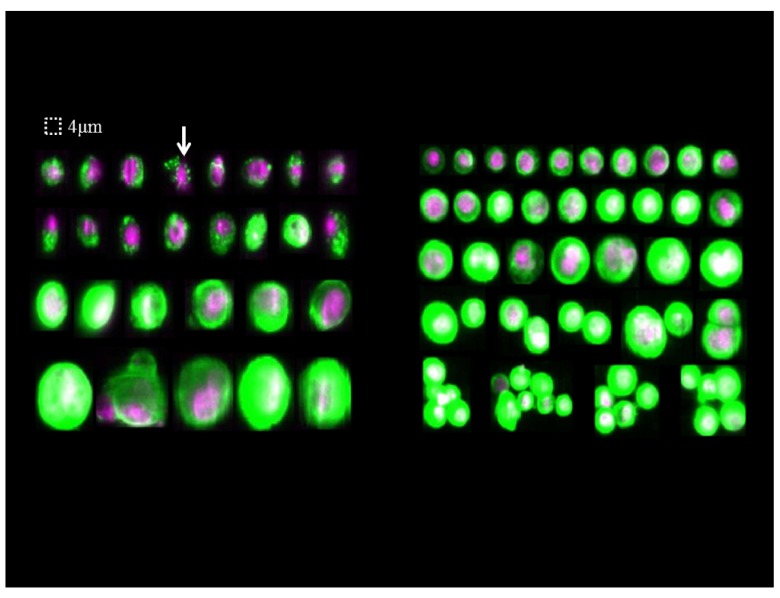
Gallery of images of tumor cells in peripheral blood (left) and ascites (right) detected by CellSearch ^®^ technology. By definition, CTCs are nucleated (purple color), express cytokeratin (green color), and lack CD45 expression. Some of the CTCs in blood sample presented morphologically apoptotic features. Arrow, shrunken cell containing CK inclusions. But in the ascitic sample, positive cells were either isolated or in clusters and their morphology was very similar to that of CTCs in the peripheral blood though without apoptotic features.

The presence of tumor cells in the peripheral blood and ascitic fluid was evaluated by the CellSearch ^®^ technology (Veridex LLC, Raritan, NJ), which detects CTCs based on ferrofluids coated with epithelial cell-specific EpCAM antibodies and a mixture of phycoerythrin-conjugated antibodies to CK 8, 18, and 19. Following informed consent, a 7.5 ml sample of peripheral blood and a 5 ml sample of ascitic fluid were collected in CellSave ^®^ tubes. The blood sample was processed in the CellTracks AutoPrep system by using the CTC kit and analyzed with the CellTracks Analyzer. For analysis of the ascitic sample, we used the method developed for the detection of tumor cells in cerebrospinal fluid samples (CSF) ^7, 8^. This methodology revealed the presence of 47 CTCs (EpCAM ^+^, CK ^+^, and CD45 ^-^) in the 7.5 ml blood sample, some with morphologically apoptotic features, and over 13,000 CTCs (EpCAM ^+^, CK ^+^, and CD45 ^-^) in the 5 ml ascitic sample. Interestingly, in the ascitic sample, positive cells were either isolated or in clusters and their morphology was very similar to that of CTCs in the peripheral blood though without apoptotic features ( Figure 3).

The patient was diagnosed with an adenocarcinoma of the lower esophagus or gastroesophageal junction, with hepatic, peritoneal and pancreatic metastases. No remission of the disease was observed under palliative treatment, and the patient died one month after diagnosis.

## Discussion

Esophageal cancer metastases are most frequently found in abdominal lymph nodes (45%), followed by the liver (35%), and lungs (25%) ^9^. Distant metastases are also seen in cervical/supraclavicular lymph nodes, bone, adrenal glands, peritoneum, brain, pericardium, pleura, stomach, and pancreas, via different pathways such as direct extension, the bloodstream, the lymphatic system or intracavitary diffusion. It has also been suggested that EAC preferentially metastasize to the liver ^9^. The case we report here is a typical esophageal adenocarcinoma with multiple metastases.

Because of the critical role of CTCs, both in the spread of cancer and in cancer research, an increasing number of studies have tried to explore technologies that enable the detection of these cells in peripheral blood. However, owing to the rarity of CTCs in this compartment, their detection requires methods combining high sensitivity and specificity. To date, there is no gold standard for CTC detection. Since 2004, CellSearch ^®^ technology has become the only FDA-approved system for the clinical detection and enumeration of CTCs in patients with metastatic breast cancer, colorectal cancer and prostate cancer ^10– 12^. This technology renders it possible to obtain highly reproducible quantitative results from different laboratories ^13^.

Currently, the results of prospective studies demonstrate that CTC enumeration has an enormous potential to estimate cancer prognosis, monitor disease recurrence and patient response to cancer therapy and even offer new insights into the comprehension of cancer biology in various epithelial cancers ^6^.

Few studies have yet documented the presence of CTCs in metastatic esophageal cancer. Allard *et al.* reported that CTCs were detected in patients with various types of carcinomas, including gastrointestinal cancers, although the median number of CTCs in gastrointestinal carcinomas was relatively low ^14^.

In 2008, Hiraiwa *et al.* studied the utility of CTC enumeration in gastrointestinal cancer patients ^15^. Thirty eight patients with esophageal cancer were included, including 23 patients with metastases. They found that 21.7% of the metastatic esophageal cancer patients had ≥2 CTCs in peripheral blood samples. Two or more CTCs in patients with metastatic esophageal cancer significantly correlated with pleural or peritoneal dissemination and a significantly worse prognosis. Therefore, the authors hypothesized that the detection of ≥2 CTCs was an important prognostic factor for metastatic esophageal cancer.

Abdominal paracentesis is an important tool in patient diagnosis and treatment, and ascitic fluid cytology is a traditional method to establish the etiology of the effusion, especially when malignancy is suspected. However, the most important problem with conventional peritoneal cytology is its lack of sensitivity and high operator-dependence. The overall sensitivity of conventional cytology for malignant ascites is 57%–67% ^16^, and the sensitivity for peritoneal carcinomatosis is 96.7%, while it is much lower (6.7%–13.3%) in ascites due to hepatocellular carcinoma, liver metastases, or chylous ascites with lymphoma ^17^. To establish an accurate diagnosis and study of tumor cells, immunocytochemistry and further genetic and molecular techniques have been proposed, such as fluorescent *in situ* hybridization (FISH), comparative genomic hybridization (CGH) and PCR-based techniques. However, most results are qualitative, even though quantitative results might be relevant, particularly for the follow-up of either local or general therapies.

Previous studies ^6, 10– 12, 14, 15^ have shown that CellSearch ^®^ technology has shown its advantages in the detection of CTCs in peripheral blood, showing that it can achieve accurate diagnosis as well as molecular analysis at the same time. To our knowledge, no previous study has reported the use of this technology for the detection of tumor cells in ascitic fluid samples.

We have recently documented a new method to identify and quantify malignant cells in the CSF using the CellSearch ^®^ technology ^7, 8^, suggesting its potential usefulness for follow-ups and treatment evaluations with cancer patients. As described here, this new method makes it also possible to detect and quantify the presence of tumor cells, either isolated or associated as microclusters in ascitic fluid, which in some studies are recognized as a “signature” of malignant metastases ^18, 19^.

Further to the description of the clinical utility of CellSearch ^®^ technology in ascitic fluid analysis, our findings raise the issue of additional potential applications. First, it may help diagnose cancer metastases with a high sensitivity and specificity. Second, an additional antibody can be added during the detection of tumor cells by the CellSearch ^®^ technology, allowing for a further biological characterization of tumor cells. Third, EpCAM+ cells can be purified by using the CTC Profile kit ^®^, making them available for a variety of applications in order to characterize their genes and/or protein expression profiles, thus providing knowledge about biological aspects of human solid tumors ^20^. This might be particularly useful in the identification of therapeutic targets and resistance mechanisms.

The case presented here confirmed the hematogenous dissemination of esophageal cancer by the detection of CTCs in the patient’s peripheral blood, and is the first report concerning the quantification of a much higher load of tumor cells in an ascitic sample. This suggests that tumor cells in ascites can be studied with the CellSearch ^®^ technology, with a great potential in patients’ diagnosis, prognosis and in the understanding of cancer biology. We propose to designate these cells in the ascitic sample as ETC for “Effusion Tumor Cells”.

## Consent

Written informed consent for publication of clinical details was obtained from the patient’s spouse.
