# Supplementary material for: Case Report: Detection and quantification of tumor cells in peripheral blood and ascitic fluid from a metastatic esophageal cancer patient using the CellSearch ® technology
Source: F1000Res. 2014 Jan 15;3:12. [Version 1] doi: 10.12688/f1000research.3-12.v1 (PMC4103486; doi:10.12688/f1000research.3-12.v1)
Supplement: Peer review history

# Peer review: Referee response for version 1

**DOI:** 10.5256/f1000research.3231.r4681

**Published:** 2014-05-28

**Authors:** Hugh Barr

**Affiliations:** 1 Department of Surgery, Gloucestershire Royal Hospital, Gloucester, UK

This is an interesting study that assesses the use of detection metastatic cancer cells in ascites. This is a proof of principle which does require more patients. As an early report this is fine.

I have read this submission. I believe that I have an appropriate level of expertise to confirm that it is of an acceptable scientific standard.

# Peer review: Referee response for version 1

**DOI:** 10.5256/f1000research.3231.r3776

**Published:** 2014-03-06

**Authors:** Eva Obermayr

**Affiliations:** 1 Department of Obstetrics and Gynecology, Medical University of Vienna, Vienna, Austria

The authors provide an interesting study on the detection of CTCs in the blood and ascitic fluid from a patients with advanced epithelial esophageal cancer using CellSearch. The study is innovative in terms of the application of the CellSearch detection for tumor cells in the ascites, nevertheless, it is not surprising that the number of tumor cells in the ascites is by far higher than in the peripheral blood. Moreover, the clinical value of the detection of tumor cells in the ascites is unclear, especially for patients with advanced disease. In future, further investigations which include the characterization of these tumor cells in terms of EMT markers will be necessary.

I have read this submission. I believe that I have an appropriate level of expertise to confirm that it is of an acceptable scientific standard.
